# Dual oxidase 1 is dispensable during *Mycobacterium tuberculosis* infection in mice

**DOI:** 10.3389/fimmu.2023.1044703

**Published:** 2023-03-03

**Authors:** Tuhina Gupta, Demba Sarr, Kayla Fantone, Nuha Milad Ashtiwi, Kaori Sakamoto, Frederick D. Quinn, Balázs Rada

**Affiliations:** ^1^ Department of Infectious Diseases, College of Veterinary Medicine, University of Georgia, Athens, GA, United States; ^2^ Department of Pathology, College of Veterinary Medicine, University of Georgia, Athens, GA, United States

**Keywords:** *Mycobacterium tuberculosis*, antimicrobial, *Duox1*, inflammation, epithelium

## Abstract

**Introduction:**

*Mycobacterium tuberculosis* (Mtb) is the primary cause of human tuberculosis (TB) and is currently the second most common cause of death due to a singleinfectious agent. The first line of defense against airborne pathogens, including Mtb, is the respiratory epithelium. One of the innate defenses used by respiratory epithelial cells to prevent microbial infection is an oxidative antimicrobial system consisting of the proteins, lactoperoxidase (LPO) and Dual oxidase 1 (Duox1), the thiocyanate anion (SCN-) and hydrogen peroxide (H2O2), which together lead to the generation of antimicrobial hypothiocyanite (OSCN-) in the airway lumen. OSCN- kills bacteria and viruses in vitro, but the role of this Duox1-based system in bacterial infections in vivo remains largely unknown. The goal of this study was to assess whether Duox1 contributes to the immune response against the unique respiratory pathogen, Mtb.

**Methods:**

Duox1-deficient (Duox1 KO) and wild-type (WT) mice were infected with Mtb aerosols and bacterial titers, lung pathology, cytokines and immune cell recruitment were assessed.

**Results and discussion:**

Mtb titers in the lung, spleen and liver were not different 30 days after infection between WT and Duox1 KO mice. Duox1 did not affect lung histology assessed at days 0, 30, and 90 post-Mtb infection. Mtb-infected Duox1 KO animals exhibited enhanced production of certain cytokines and chemokines in the airway; however, this response was not associated with significantly higher numbers of macrophages or neutrophils in the lung. B cell numbers were lower, while apoptosis was higher in the pulmonary lesions of Mtb-infected Duox1 KO mice compared to infected WT animals. Taken together, these data demonstrate that while Duox1 might influence leukocyte recruitment to inflammatory cell aggregates, Duox1 is dispensable for the overall clinical course of Mtb lung infection in a mouse model.

## Introduction

Tuberculosis (TB) caused by *Mycobacterium tuberculosis* (*Mtb*) continues to be a major global disease annually affecting millions worldwide. An estimated 10 million people developed active TB in 2018, with 1.2 million deaths among HIV-negative people and an additional 251,000 deaths among HIV-positive individuals (1). There are no effective vaccines against pulmonary TB in adults and no biomarkers for protective immunity (2–7). While there are drugs that are effective against *Mtb*, because of the long-term treatment regimen, patient non-compliance is common leading to development of drug-resistant strains (4).

The earliest encounter between *Mtb* bacteria and the host occurs at the interface between innate immune cells and the bacilli (8). While innate immunity is critical for early anti-mycobacterial responses, it is also important for the progression of infection and long-term control of *Mtb*. It has long been recognized that immunity to *Mtb* can provide both protection and cause tissue damage (8). The pathogenesis of TB begins with inhalation of the bacilli and their interaction with cells in the respiratory tract, and is ultimately mediated by responses of the innate and adaptive immune systems (9, 10). The different steps involve macrophages, dendritic cells, pathogen-associated molecular patterns (PAMPs), pattern recognition receptors (PPRs), cytokines, and T cells, as well as neutrophils and monocytes (8–10).

Additionally, several *Mtb* gene products are known to interact with airway epithelial cells – heparin-binding hemagglutinin (HBHA) and Rv3351c (11, 12). HBHA is a major adhesin molecule involved in binding of *Mtb* to the epithelial cells, with ultimate necrosis of these cells likely contributing to extrapulmonary dissemination to various organs. Additionally, our group demonstrated that *Mtb* Rv3351c is involved in maximally effective entry and trafficking within alveolar epithelial cells (12). However, the role of epithelial cells in innate immunity against *Mtb* is at an early stage of investigation and the involvement of components of the airway surface liquid (ASL) has mostly been ignored. It is known that upper airway epithelial cells are inert to direct *Mtb* interaction; however, they release several chemokines and defensins that likely help modify the local environment along with infected alveolar macrophages (13).

Bronchial epithelial cells orchestrate an oxidative extracellular antimicrobial system present in the ASL consisting of the protein lactoperoxidase (LPO), the thiocyanate anion (SCN^-^), and hydrogen peroxide (H_2_O_2_) generated by the NADPH oxidase enzyme Dual oxidase 1 (Duox1) [reviewed by (14)]. LPO oxidizes SCN^-^ into microbicidal hypothiocyanite (OSCN^-^) in the presence of H_2_O_2_ killing a wide variety of viruses, bacteria, fungi and parasites *in vitro* (15–19). *Mtb* has not been investigated yet for its *in vitro* susceptibility to OSCN^-^. We have recently shown the relevance of Duox1 in antiviral innate immunity in a murine model of influenza infection (20). Mice with global Duox1 deficiency had significantly higher susceptibility to influenza A lung infection than wild-type animals. Our recent *in vivo* investigations have shown that both SCN^-^ and LPO are present in the airway surface liquid of both *Duox1* KO and WT mice (20). This antimicrobial system has long been characterized *in vitro*, but its relevance *in vivo* has remained unknown until recently. Because DUOX1 is highly expressed by bronchial epithelial cells and is the main H_2_O_2_ provider for the LPO/H_2_O_2_/SCN^- -^ antimicrobial system (21–24), we hypothesized that *Duox1* KO mice will have higher bacterial loads and more severe lung pathology than wild-type (WT) animals following *Mtb* infection.

Our data indicate that both *Duox1* KO and WT mice have significantly higher inflammation in the lung tissue and airways at 4 weeks post-infection upon *Mtb* infection compared to mice at day 1 post-*Mtb* infection. Surprisingly, Duox1 does not have a protective role during *Mtb* infection *in vivo*. These data indicate that Duox1 is overall dispensable in *Mtb* infection *in vivo*.

## Materials and methods

### Mice

All mouse procedures were approved by the Animal Care and Use Committee at The University of Georgia (UGA). *Duox1* KO and C57BL/6J (WT) mouse colonies were maintained in the Coverdell vivarium at The University of Georgia. The *Duox1*-deficient mice were established on a B6 background by retroviral-based gene-trapping methodology (25, 26) by Lexicon Pharmaceuticals. We thank the company and Miklós Geiszt (Semmelweiss University, Budapest, Hungary), the sole academic distributor, for providing the *Duox1* KO mouse strain. Mice were housed with a standard diet and given water add libitum in a specific pathogen-free facility, according to The University of Georgia Animal Use Protocol Guidelines.

One week before infection, mice were transferred to the Animal Health Research Center, an animal biosafety level 3 (ABSL-3) facility, in order to perform aerosolized *Mtb* infections. Both male and female mice between the ages of 6 to 12 weeks were used in the study. All animals were housed with 12-hour light/dark cycle, *ad libitum* mouse chow and water, and provided environmental enrichment. Animals were examined at least daily for signs of distress and discomfort using a scoring system (weight loss 10–20% = 1 point, cyanosis = 1point, mild lethargy = 1 point, hunched back = 1 point, weight loss > 20% = 3 points, dyspnea = 3 points) in which any animal accumulating 3 points is euthanized.

### Mtb culture and infection

The seed stocks of *Mtb* strain Erdman were cultured as pellicles in Proskauer Beck broth medium. Oleic Albumin Dextrose Catalase enrichment was used in the agar plates. The working *Mtb* stocks used to infect mice were prepared as described previously (27). A stock was used as an inoculum to infect mice with *Mtb* aerosols generated within a Madison chamber. This chamber has been calibrated to deliver 200 CFU of nebulized bacteria to the lung of the mice during a 15-minute exposure window as previously described (27). The Madison chamber holds 18 cages of mice in a carousel, and all animals per experiment from each genotype (WT and *Duox1* KO) were randomly dispersed in the carousels in one single run to control for possible variability of *Mtb* exposure within the Madison chamber. Each experiment included 15 males and 15 females from each genotype.

### Euthanasia and tissue collection

At 1, 30, and 90 days post-infection, mice were euthanized by CO_2_ asphyxiation, and blood was collected by cardiac puncture. Bronchoalveolar lavage (BAL) fluid was also collected using 1 ml of sterile phosphate-buffered saline (1X PBS). These samples were stored at 4°C until assayed. The lungs were perfused with sterile saline. Whole lungs, spleen and mediastinal lymph nodes (MLN) were collected in RPMI and kept at 4°C until lung leukocytes were isolated and stained. From another set of animals, a portion of the lung (right lobe) was collected (equal number of males and females from each genotype) to determine bacterial load (CFUs) and another piece (left lobe) was fixed with 10% neutral-buffered formalin for histology. Liver and spleen were also collected to determine dissemination and trachea was fixed in 10% buffered formalin for histology and further analysis by immunofluorescence and/or immunohistochemistry.

### Enumeration of bacterial load

Lung, liver and spleen from infected mice were aseptically collected in Whirl-Pak bags (Cat# B01062, Nasco, VWR, Radnor, PA) and frozen at -80°C. For plating, the tissues were resuspended in sterile 1X PBS and hand-homogenized. Serial dilutions of the lysates were plated on Middlebrook 7H11 agar plates supplemented with 0.5% glycerol, 10% albumin, dextrose and saline (ADS), 0.05% Tween 80 (7H11gtADS) and incubated at 37°C for 3-6 weeks. The colonies were counted and bacterial load in each organ calculated and graphed on a log10 scale.

### Lung single cell suspension

The lungs were chopped in Petri dishes with a sterile scalpel and incubated at 37°C with a digestion medium containing collagenase (Cat#07912, Stemcell Technologies, Cambridge, MA) and DNAse I (Cat# 07900, StemCell Technologies, Cambridge, MA) in a shaking platform. Cell suspensions were passed through a 70 µm nylon mesh strainer before counting and staining with labeled antibodies.

### Spleen and mediastinal lymph node single cell preparation

Single cell suspension of the spleen and MLN were prepared by passing hand-homogenized tissues through a 70 µm nylon mesh. The cells were spun at 350 x g for 10 minutes, the cell pellet was resuspended in ACK lysing buffer (Cat# 10-548E; Lonza, Walkersville MO) for 30 seconds. The cells were washed with 1X PBS, resuspended, counted and stained with a viability dye followed by labeled antibody cocktail as above.

### Cytokine quantification

The Bio-Plex Pro Mouse Chemokine Panel 31-plex magnetic bead-based assay was used to measure levels of 31 different mouse cytokines and chemokines in *Mtb*-infected, cell-free BAL fluid (collected on 1 and 30 day post infection, centrifuged, and stored at -80°C as indicated above) using internal standards (Cat#:12009159, Bio-Rad, Hercules, CA) following manufacturer’s instructions and as previously described (20, 28). Samples were read with the Bio-Plex 3D analyzer (Bio-Rad Inc., Hercules, CA) and data were exported to Excel before statistical analysis.

### Flow cytometry

The antibodies used here are listed in **Table 1**. BAL cells, splenocytes, MLN and lung single cell suspensions were washed once in 1X PBS, and leukocytes were resuspended in 1 ml sterile 1X PBS, diluted when necessary and counted with an automatic cell counter Countess TM Automated Cell Counter, Invitrogen. For viability measurement, 1X10^6^ cells were suspended in 100 µl of 1X PBS and stained first with Zombie Aqua™ flexible viability dye (Cat#: 423101; BioLegend, San Diego, CA) at room temperature in the dark for 15 minutes. Following a washing step, the cells were resuspended in 100 µl of 1X PBS containing 1% bovine serum albumin and blocked using TruStain FcX™ anti-mouse (CD16/32) antibody (Cat#: 101320; BioLegend, San Diego, CA) on ice and protected from light for 10 minutes. Multicolor cell surface staining was performed separately using two different antibody cocktails for the characterization of lymphoid (CD45, CD3, CD4, CD8, and NK1.1) and myeloid cells (CD11b, CD11c, F4/80, Ly6G, Ly6C, and CD115) as previously described (20, 28, 29). The cells were subsequently stained with surface antibodies (**Table 1**) at a 1:100 dilution at the same time to detect the number of cells from each of the myeloid and lymphoid cell subsets following the protocol as described previously (20). Samples were read within 12 hours using the NovoCyte Flow Cytometer (Agilent Technologies, Santa Clara, CA) with the NovoExpress^®^ software at the University of Georgia College of Veterinary Medicine Cytometry Core Facility. Data acquisition was followed by analysis using FlowJo v.10.8.1 (BD Biosciences, San Jose, CA) with high-dimensional data analysis algorithms using FlowJo plugins. Flow data were also analyzed using a previously optimized gating strategy for identification of the cell populations of interest (20). Myeloid cell clustering was also assessed by uniform manifold approximation and projection (UMAP) analysis.

**Table 1:** Summary table of the antibodies used for multicolor flow cytometry in this study and the gating strategies that were applied for the data analysis.

				Lymphoid cells			
Antibodies or dye used	Clone	Source	Cat#	Live cells	T helper	Cytotoxic T cells	NK cells
Zombie Aqua		Biolegend	423101	**-**	**-**	**-**	**-**
CD45 AF700	30F11		103125		**+**	**+**	**+**
CD3 APC	17A2		100235		**+**	**+**	**-**
CD4 AF488	GK1.5		100425		**+**	**-**	**-**
CD8 APCF750	53-6.7		100765		**-**	**+**	**-**
NK1.1 PE	PK136		108707		**-**	**-**	**+**
				Myeloid cell population 1			
Antibodies or dye used	Clone	Source	Cat#	Live	Neutro.	Macro.	Alv. macro
Zombie Aqua		Bioledgend	423101	**-**	**-**	**-**	**-**
CD11b PECY7	M1/70		101215		**+**	**+**	**-**
CD115 APC	AFS98		135509		**-**		
Ly6G AF488	1A8		127625		**+++**		
Ly6C APCF750	HK1.4		128045				
F4/80 PE	BM8		123109			**+**	**+**
CD11c BV605	N418		117333				**+**
	Myeloid cell population 2						
	gMDSCs	mMDSc	Eosino.	Dcs	Mono	In. Mono	Inf. macro
Zombie Aqua	**-**	**-**	**-**	**-**	**-**	**-**	**-**
CD11b PECY7	**+**	**+**	**+**	**+**	**+**	**+**	**+**
CD115 APC			**-**	**-**	**+**	**+**	
Ly6G AF488	**+**		**-**		**+**		
Ly6C APCF750		**+**	**-**			**+++**	
F4/80 PE			**+**	**-**			**+**
CD11c BV605			**-**	**+**			**-**

Cat#, Catalog number; NK, natural killer cells; Neutro., neutrophils; macro., macrophages; Alv. Macro., Alveolar macrophages; DCs, dendritic cells; gMDSCs, granulocyte myeloid-derived suppressor cells; mMDSCs, monocytic myeloid-derived suppressor cells; Eosino, eosinophils; Mono., Monocytes; Inf. Mono., inflammatory monocytes; Inf. Macro, Inflammatory macrophages. Data for gMDSCs, mMDSCs, as well as eosinophils are not shown.

### Histopathology

*Duox1* KO and WT mice were sacrificed as indicated above. Lungs were injected with 1 ml of 10% neutral-buffered formalin (NBF) and stored in 10% NBF. Formalin-fixed, paraffin-embedded lung tissue blocks were prepared by the Histology Laboratory at the University of Georgia College of Veterinary Medicine. Lung tissue sections (5-µm thickness) were either left unstained for subsequent immunofluorescence or immunohistochemistry or stained with hematoxylin and eosin (H&E) stains for histopathology. Histopathology was performed by a board-certified, veterinary pathologist who was blinded to the mouse genotype and experimental conditions. In addition to the number of inflammatory cell aggregates (“granulomas”) histologically distinguishable in the section, the percent of affected lung tissue was estimated. Scoring for alveolar infiltrates, alveolar edema, necrosis, and pleuritis was on a scale of 0 to 4 based on distribution (0 = no lesions, 1 = focal, 2 = multifocal, 3 = coalescing, 4 = diffuse). The neutrophil score was based on percentage of neutrophils within alveolar infiltrates (0 = no neutrophils, 1 = up to 25% of cells, 2 = 26-50%, 3 = 51-75%, 4 = 76-100%). The perivascular cuffing (PVC) score was based on thickness of leukocytes around blood vessels (0 = no cuffing, 1 = 1 layer of cells, 2 = 2-5 layers, 3 = 6-10, 4 = greater than 10). The vasculitis score was based on severity of the lesion (0 = no inflammation, 1 = vessel wall infiltrated by cells, 2 = smooth myofiber separation and cellular infiltration, 3 = changes in 2 + fibrinoid change, 4 = effacement of the vessel wall). The interstitial pneumonia score was based on thickness of alveolar septa (0 = no infiltration, 1 = 1 leukocyte thickness, 2 = 2 leukocytes thick, 3 = 3 leukocytes thick, 4 = 4 leukocytes thick).

### Immunofluorescence

Unstained lung tissue sections were deparaffinized and rehydrated with a xylene and alcohol gradient. Sections were antigen-retrieved with 0.1 M sodium citrate in a Pascal Pressure Cooker (Agilent Technologies, Santa Clara, CA) for 20 minutes at 95°C, and then permeabilized with 0.1% Triton X-100 while blocking in PBS with 5% BSA and 10% normal horse serum for 1 hour. Primary antibody was used for detection of *Mtb* bacteria (cat# NR-13820; BEI Resources, Manassas, VA). In some cases, anti-CD45R (Cat#: 550286, BD Biosciences, San Jose, CA) anti-CD68 (Cat#: ab955; Abcam; Waltham MA) and anti-cleaved caspase-3 (Cat#: 9661, Cell Signaling Technologies, Danvers, MA) antibodies were also applied to assess B cells and apoptosis, respectively. In each case, the primary antibody dilution was optimized, and all dilutions and antibody incubations were done in 1X TBST with 0.5% BSA, 1% normal horse serum, and 0.01% Triton X100 overnight at 4°C. Sections were washed the very next morning using 1X TBST, and secondary antibody staining was performed at a 1:1,000 dilution in 1X TBST with 0.05% BSA, 0.1% normal horse serum for 1 hour with horse anti-rabbit IgG antibody (H+L) conjugated with FITC (Vector Laboratories, cat#: FI-2000-1.5). Sections were washed with 1X-TBST, and then Vectashield™ anti-fade mounting medium with DAPI (Cat#: H-1200-10, Vector Laboratories, Burlingame, CA) was applied to sections before adding the coverslip. All digital images were acquired on a Nikon A1R confocal microscope (Nikon Eclipse Ti-E inverted microscope) and examined with NIS Element software (Nikon, Version 6.4). Fluorescence intensity was quantified using ImageJ (NIH, version 1.8.0), and data were exported to Excel and analyzed for statistical significance and plotting using Graph Pad Prism 9.2.0. 3-5 sections per mouse were analyzed.

### Immunohistochemistry

The expression pattern of several host proteins in the mouse lung was also assessed by immunohistochemistry using the following primary antibodies: anti-cleaved caspase 3 (Cat# 9661, Cell Signaling, Danvers, MA), anti-myeloperoxidase (Cat# PA5-16672; Invitrogen, Waltham, MA), anti-neutrophil elastase (Cat# PA5-87158; Invitrogen, Waltham, MA), and anti-CD45R (Cat: 550286; BD Pharmingen; San Diego, CA).

The unstained sections of lung tissues from *Mtb*-infected WT and *Duox1* KO mice were deparaffinized and rehydrated as above. After antigen-retrieval, endogenous peroxidase blocking was performed by incubating tissue sections with Dual Endogenous Enzyme Block (Cat# S2003, Agilent Technologies, Santa Clara, CA). Sections were permeabilized in 1X TBST containing 0.5% Triton X-100 for 5 minutes followed by blocking in Rodent Block (Cat# RBM961, Biocare Medical, Pacheco, CA). Tissue sections were then incubated in primary antibodies overnight followed by appropriate Polymer HRP-conjugated secondary antibodies (Cat# D13-18 for rabbit primary antibody or D13-52 for mouse primary antibody, GBI Labs Golden Bridge International, Bothell, WA) followed by a 5-minute 3,3′-Diaminobenzidine (DAB) treatment. Hematoxylin (Cat# H3401, Vector Laboratories, Newark, CA) and Acrytol Mounting Medium (Cat# 13158, EMS, Hatfield, PA)), were used for counterstaining and mounting, respectively. DAB signal quantification was done using ImageJ software (NIH, Bethesda, MA).

### Myeloperoxidase and neutrophil elastase ELISA

Using BAL fluid, sera and lung lysates from WT and *Duox1* KO mice, we quantified MPO and NE using DuoSet ELISA kits (MPO; Cat# 3667; NE; Cat#4517-05, R&D Systems, Minneapolis, MN) following the manufacturer’s instructions. Primary antibody against MPO (800 ng/ml) or NE (1 μg/ml) was added to 96-well plates overnight at room temperature. Plates were washed, samples and recombinant protein standards (ranging from 250-16,000 pg/ml for MPO and 12.5-800 pg/ml for NE) were added to the plates overnight at 4°C. Plates were again washed and detection antibodies were added for 2 hours at room temperature at concentrations of 50 ng/ml for anti-MPO and 100 ng/ml of anti-NE, respectively. Following incubation, the plates were washed and streptavidin-HRP was added for 20 minutes at room temperature at a 200-fold dilution for MPO and 40-fold dilution for NE. Substrate solution was added at a 1:1 mixture of H_2_O_2_ and tetramethylbenzidine. Once the reaction was complete, 1N HCl was added to stop the reaction and absorbance at 450 nm was used to quantify the respective protein in pg/ml.

### Neutrophil elastase activity assay

Enzymatic activity of NE was evaluated using the NE activity assay kit (Cat#600610; Cayman Chemical; Ann Arbor, MI) as previously described (28). Neutrophil elastase at 18 mU/ml was used to make a standard curve ranging from 0.156 mU/ml to 10 mU/ml. Standards and samples were added to a black, flat-bottom, 96-well plate. Ten μl of substrate solution consisting of Cell-Based Assay DMF and (Z-Ala-Ala-Ala-Ala)2Rh110 was added to each standard and sample, which is selectively cleaved by elastase to yield the highly fluorescent compound R110. Fluorescence was analyzed with an excitation wavelength of 485 nm and an emission wavelength of 525 nm using a microplate fluorimeter (Varioskan Flash, Thermo Scientific). Kinetic fluorescent measurements were taken every 2 minutes for 2 hours at 37°C. Average fluorescence was taken for each well and quantified in mU/ml with the help of the NE standard.

### Statistical analysis

CFU data were log_10_ transformed before statistical analysis. GraphPad Prism Software 9.2.0 was used to carry out all statistical analysis. Quantified data were either analyzed between groups with unpaired Student’s *t* test or Mann-Whitney U test as appropriate. In some quantified data, statistical analysis required One-way ANOVA with Tukey’s *post-hoc* multiple comparison (for comparing three or more groups). Data are expressed as mean ± S.E.M. where *p ≤ 0.05; **p ≤ 0.01; ***p ≤ 0.001; ****p ≤ 0.0001 indicates the level of significance. Relevant information, such as test used, the number of animals/samples, and the *p* values obtained, are given for each figure in the figure legend.

## Results

### *Mtb* growth *in vivo* is Duox1-independent

**Figure 1:**
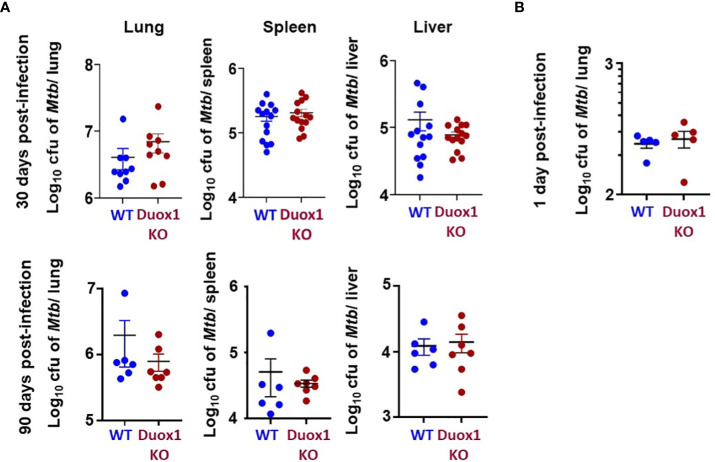
*Mtb* growth *in vivo* in *Duox1* KO mice. Mature *Duox1* KO and WT mice were aerogenically infected with 200 CFU of *Mtb* (Erdman). **(A)** On 30 and 90 days post-*Mtb* infection, mice from each genotype were euthanized and lungs (n=9), spleens (n=14) and livers (n=14) were removed for CFU burden assessment by colony counting. **(B)** On 1 day post-infection, lungs from mice (n=5) of each genotype were removed for CFU counts. Mann-Whitney U test was used to compare CFUs between animal groups. Mean+/-S.E.M. CFU, colony-forming unit; *Duox1* KO: *Duox1*-deficient; WT, wild-type.

**Figure 2:**
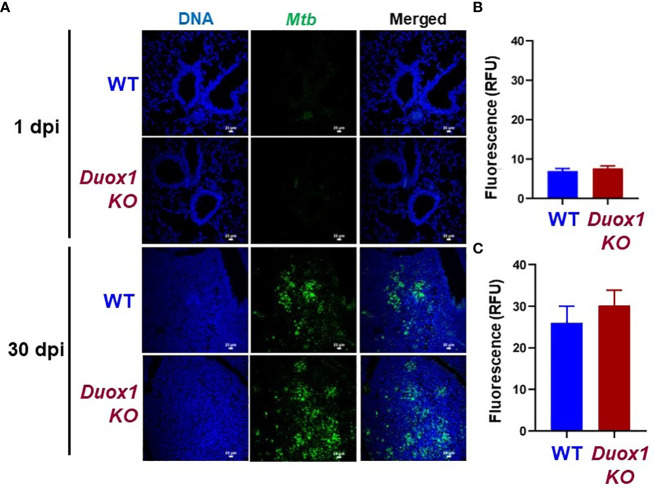
Duox1 does not affect *Mtb* proliferation in the lung. Lungs from *Mtb*-infected *Duox1* KO and WT mice were harvested at 1 and 30 day(s) post-infection, respectively. Fixed and processed lung tissues were stained with primary anti-*Mtb* antibody followed by a secondary AF488 antibody. **(A)** Representative fluorescent images of six similar results are shown. White scale bars indicate 25 μm. Mean green fluorescent intensities (MFI) indicative of *Mtb* burden were quantified for **(B)** 1 day post-infection and **(C)** 30 days post-infection in *Duox1* KO (n=6) and WT mice (n=6). Error bars represent ± SEM. Mann Whitney U-test. *Duox1* KO: *Duox1*-deficient; WT, wild-type; RFU, relative fluorescence unit.

In our previous study we showed that wild-type mice express Duox1 in their airway epithelium and have elevated H_2_O_2_ levels in their bronchoalveolar lavage compared to *Duox1*-deficient animals (20). To evaluate the effect of a functional *Duox1* on *Mtb* infection, we infected male and female *Duox1* KO and C57BL/6J (WT) mice. Following *Mtb* aerogenic infection, bacterial colony forming units (CFUs) at 1, 30, and 90 days post-infection were measured in the lung, liver and spleen to evaluate bacterial growth and dissemination. Our results show that, compared to WT mice, CFUs in *Mtb*-infected *Duox1* KO mice were not significantly different in the lung, spleen or liver at days 1, 30 or 90 post-infection, respectively (**Figures 1A, B**). To further evaluate the effects of Duox1 on bacterial growth inside the lung, *Mtb*-infected WT and *Duox1* KO mouse lungs were sectioned and stained with a polyclonal anti-*Mtb* antibody. Bacteria were not detected inside of the lung tissue at 1 day post-infection, as expected, in both WT and *Duox1* KO animals (**Figure 2A**). However, at 30 days post-infection, bacteria (green) are present in inflammatory cell aggregates (**Figure 2A**). Quantification of green fluorescence indicative of *Mtb* burden did not yield significant differences between WT and *Duox1* KO mice neither at 1 nor 30 day(s) post-infection (**Figures 2B, C**). Despite our initial hypothesis, CFU data from the lung, spleen, and liver, and visual quantification of the bacteria inside the lungs suggest that *Mtb* lung infection does not depend on host Duox1 expression.

### Lung histopathology during *Mtb* infection is not affected by Duox1

**Figure 3:**
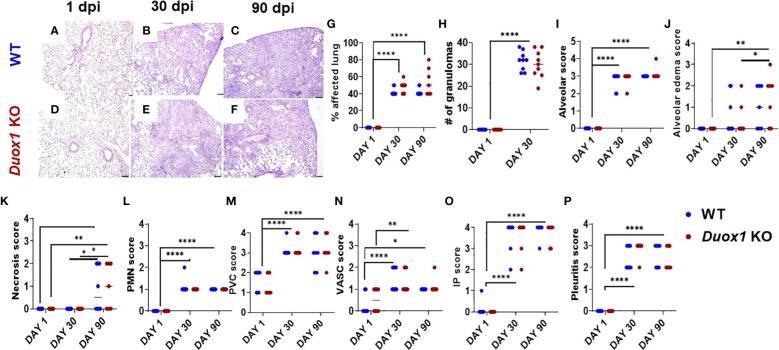
Lung pathology during *Mtb* infection is independent of Duox1. *Mtb*-infected lung lobes from both *Duox1* KO and WT mice were isolated and fixed in 10% buffered formalin at 1, 30, and 90 day(s) post-infection. Fixed lung tissues were processed for histology, sectioned, and subjected to hematoxylin and eosin staining. Lungs collected at 1 day post-infection from both mouse background **(A, D)** do not show any evidences of lung pathology while granuloma-like lesions in both groups are visible at 30 **(B, E)** and 90 **(C, F)** days post-infection. **(G)** Comparative percentage of lung tissue affected (0% to 80%). **(H)** The number of inflammatory aggregates inside the lung tissues (from 0 to 40 maximum). The lung histological score was assessed with a scale from 0 to 4 and included the **(I)** alveoli, **(J)** the alveolar edema, **(K)** necrosis, and **(L)** polymorphonuclear neutrophils as well as **(M)** perivascular score. **(N)** The vascular, **(O)** IP score and **(P)** pleuritis are also shown. Sample sizes: n=8 Duox1 KO; n=8 WT mice for histopathological scoring at 1 day post-infection. For 30 days post-infection: n=9 *Duox1* KO and n=9 WT mice were used. For 90 days post-infection, n=6 WT and n= 7 *Duox1* KO animals were assessed. ANOVA with multiple comparison was used for statistical data analysis. *p<0.05, **p<0.01, ****p<0.0001. IP, intraperitoneal, WT, wild-type; day post infection, days post-infection; PMN, polymorphonuclear neutrophil granulocyte; *Duox1* KO, *Duox1*-deficient; WT, wild-type.

Since we hypothesized that Duox1 could affect lung pathology during *Mtb* infection, lungs of *Mtb*-infected *Duox1* KO and WT mice collected at 1, 30, and 90 day(s) post-infection, were stained with hematoxylin and eosin (**Figures 3A-C** for WT and **Figures 3D-F** for *Duox1* KO mice) and examined by a board-certified veterinary pathologist blinded to the experimental groups. The percent of lung affected (**Figure 3G**), the number of “granulomas” or inflammatory cell aggregates (**Figure 3H**), the extent of alveolar infiltration (**Figures 3I, J**) and necrosis (**Figure 3K**), the neutrophil score (**Figure 3L**), perivascular infiltration (**Figure 3M**), vasculitis (**Figure 3N**), interstitial pneumonia (**Figure 3O**), and pleuritis (**Figure 3P**) were all not different between *Duox1* KO and WT mice on any of the days assessed (1, 30, 90 day(s) post-infection). However, all parameters were significantly higher at 30 or 90 days post-infection compared to 1 day post-infection, suggesting that infection was chronically established in mice, irrespective of their genetic background. Overall, the analysis suggested that Duox1 does not affect lung pathology during *Mtb* infection *in vivo*.

### Duox1 influences the cytokine environment in the lung during *Mtb* infection

A lack of any effect of Duox1 on lung pathology does not mean that Duox1 could not have a role in *Mtb* infection. To further investigate a possible *in vivo* role for Duox1 in the immune response to *Mtb* infection, pro- and anti-inflammatory cytokines/chemokines in the cell-free BAL fluid from both *Mtb*-infected WT and *Duox1* KO mice were quantified using a bioplex beads array. Duox1 expression has been detected by us previously in human macrophages and Duox1 could alter macrophage functions including proinflammatory cytokine release (30). None of the cytokines/chemokines was significantly different between WT and *Duox1* KO at 1 day post-infection (**Supplementary Figures 1**, **2**). However, at 30 days post-infection, TNF-α, IL-6, KC, CCL3, CCL2, CCL20, CXCL11, CCL11), CCL27, CXCL5, CXCL12 and CCL5 were all significantly higher in the BAL fluid of *Duox1* KO compared to WT mice (**Supplementary Figure 1**). Only CXCL16 and CCL1 were significantly higher in WT compared to *Duox1* KO mice at 30 days post-infection (**Supplementary Figure 1**), while additional cytokines/chemokines showed no significant differences between WT and *Duox1* KO mice (**Supplementary Figure 2**). Overall, our data suggest a potential role of Duox1 in shaping the localized immune response in *Mtb* infection *in vivo*.

### Immune cell recruitment to the airways during *Mtb* infection does not depend on Duox1

**Figure 4:**
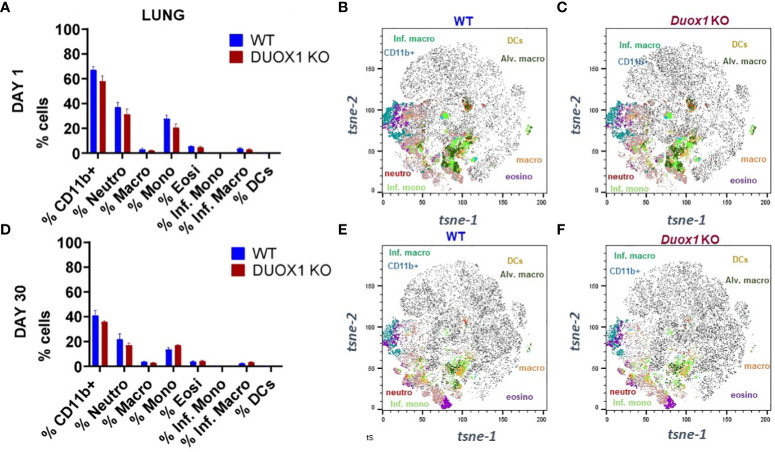
Myeloid cell populations in the lung tissues of *Mtb*-infected WT and *Duox1* KO mice. **(A)** Mean observed cell proportions determined by flow cytometry in lung lysates from two independent experiments (n= 5 per mouse genotype) of *Mtb*-infected WT mice at 1 day post-infection. **(B)** Marker expression by *t-sne* in *Mtb*-infected WT mice at 1 day post-infection. **(C-F)** Marker expressions by *t-sne* in *Mtb*-infected *Duox1* KO and WT mice at 1 and 30 day(s) post-infection as indicated. *Duox1* KO, *Duox1*-deficient; WT, wild-type.

Because Duox1 may have a role in the immune responses against *Mtb* infection *in vivo*, we next used multicolor flow cytometry to determine the phenotype of immune cells recruited in the airway and the spleen using a previously optimized protocol (20, 28). We determined previously that there are no significant differences in the nature of leukocytes in the airway of uninfected, naïve, WT and *Duox1* KO mice (20). The protocol used markers that identifiy lymphoid and myeloid cell subsets. Overall, our data show that there are no significant differences in the percent of cells in the spleen, lung, BAL, and mediastinal lymph node (MLN) for both major lymphoid and myeloid cell subsets in *Mtb*-infected WT versus *Duox1* KO animals (**Figures 4A, D**; **Supplementary Figures 4**, **5**, **6A, D**). To further analyze the multicolor flow data, we used high-dimensional algorithms with FlowJo plugins. All data were analyzed by t-sne analysis (**Figure 4** and **Supplementary Figures 4**-**6B, C, E F**) while lung myeloid cell populations were also clustered by uniform manifold approximation and projection (UMAP) for comparison (**Supplementary Figure 3**). Gating overlay of lymphoid cell subsets in *t-sne* dimension 1 and 2 indicated lower density of CD4 and CD45 clusters in the spleen (**Supplementary Figures 4E, F**) at 30 days post-infection, while the density of clusters remained identical for WT and *Duox1* KO at 1 day post-infection (**Supplementary Figures 4B, C**). However, the density of CD4+ cells inside the T cell cluster is lower in *Duox1* KO at 30 day post infection (**Supplementary Figure 3F**) compared to WT (**Supplementary Figure 4E**). Like lymphoid cells, the myeloid cell clusters in the spleen also show different clustering, especially for neutrophils (**Supplementary Figures 5E, F**). However, these relative differences are not seen in the entire lung single cell suspension at neither 1 (**Supplementary Figures 5B, C**), nor 30 day(s) post-infection (**Supplementary Figures 5E, F**). In the BAL and mediastinal lymph node (MLN), no apparent differences between myeloid clusters were observed (**Supplementary Figures 6B, C, E, F**). Overall, Duox1 does not significantly affect the immune cell composition in the studied organs.

### Airway levels of neutrophil markers increase during *Mtb* infection in a Duox1-independent fashion

**Figure 5:**
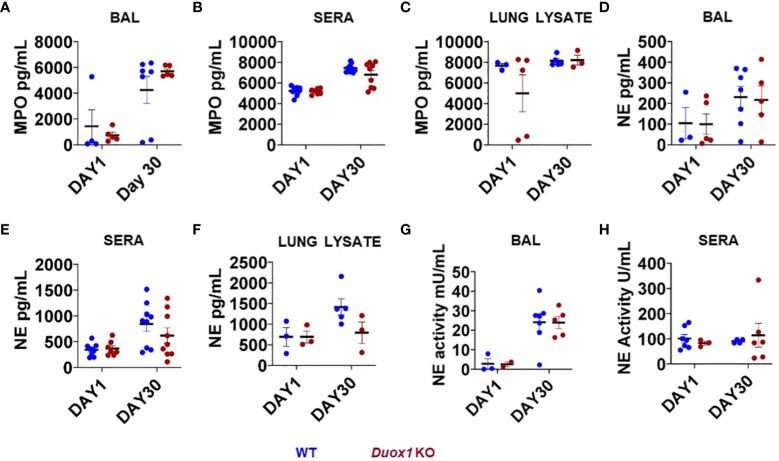
Neutrophil markers in the blood and airways of *Mtb*-infected *Duox1* KO mice. The concentrations of MPO and NE were measured in the BAL fluid, sera and lung lysates from *Mtb*-infected WT and *Duox1 KO* mice at 1 and 30 days post-infection. NE enzymatic activities in cell-free BAL fluid and sera are also shown. The following parameters were measured at 1 and 30 day(s) post-infection. **(A)** BAL MPO, **(B)** serum MPO, **(C)** MPO in lung lysates, **(D)** BAL NE, **(E)** serum NE, **(F)** NE in lung lysates, **(G)** NE activity in BAL and **(H)** NE activity in sera. Statistical comparisons were done with ANOVA followed by multiple comparisons. No significant differences were found. The concentrations of both MPO and NE are expressed in pg/ml. BAL, bronchoalveolar lavage fluid; *Duox1* KO, *Duox1*-deficient; MPO, myeloperoxidase; NE, neutrophil elastase; WT, wild-type.

Phagocytic cells (i.e., neutrophils and macrophages) are the predominant “warriors” of *Mtb*-induced inflammatory responses that play important roles in immune defense but also lung pathology (31, 32). Macrophages act as the first line of defense against *Mtb* inside the lung (33). The heme protein myeloperoxidase (MPO) is an active major component of neutrophils that can also be found at lower quantities in monocytes and inflammatory macrophages (34, 35). In the presence of H_2_O_2_, MPO generates hypochlorous acid (HOCl) using chloride (35). We have shown that naïve, uninfected *Duox1* KO mice have significantly lower levels of H_2_O_2_ in their BAL fluid than WT mice (20). To assess a possible Duox1-dependent change in MPO levels following *Mtb* infection, we collected BAL fluids, sera and lung homogenates from *Mtb*-infected *Duox1* KO and WT mice at 1 and 30 day(s) post-infection. Our data show that MPO increases, mainly in the BAL, over time with *Mtb* infection, but the differences between *Mtb*-infected WT and *Duox1* KO mice were not statistically significant in the BAL (**Figure 5A**), sera (**Figure 5B**), or lung lysates (**Figure 5C**).

**Figure 6:**
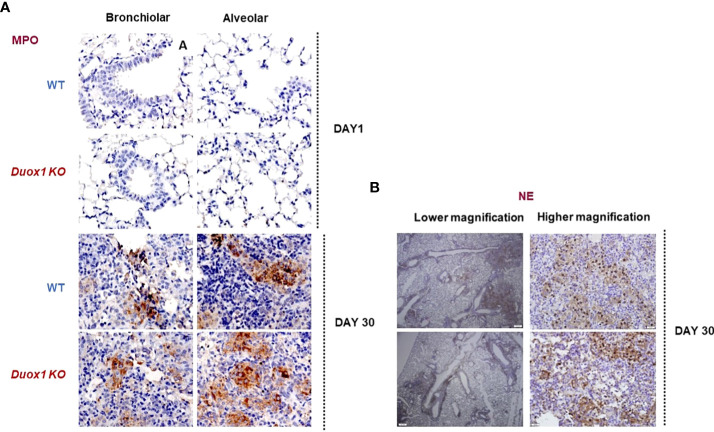
MPO and NE expressions in the lungs of *Duox1* KO mice infected with *Mtb*. *Mtb*-infected lungs from *Duox1* KO and WT mice were harvested at 1 or 30 day(s) post-infection, fixed and processed for staining with rabbit anti-MPO or rabbit anti-NE antibodies followed by anti-rabbit secondary antibody (HRP-linked) and DAB amplification. Sections are counterstained with hematoxylin and representative micrographs are shown in panels. **(A)** Representative micrograph of MPO staining of *Mtb*-infected lungs (bronchiolar area) of *Duox1* KO and WT mice at 1 and 30 day(s) post-infection. **(B)** Representative photomicrographs of NE in *Mtb*-infected lungs at 30 days post-infection, at low (200 µm) and high magnification (20 µm), respectively. *Duox1* KO, *Duox1*-deficient; MPO, myeloperoxidase; NE, neutrophil elastase; WT, wild-type.

Neutrophil elastase (NE) is a major protease released from neutrophils during airway inflammation. A previous study has demonstrated that Duox1 is required for neutrophil recruitment in a mouse model of allergic asthma (36). To address whether NE release inside *Mtb*-infected airways is affected by Duox1, NE concentrations were assessed by ELISA in the BAL fluid, sera, and lung lysates of *Mtb*-infected WT and *Duox1* KO mice as above (**Figures 5D–F**). In addition, NE enzymatic activity was measured as previously described (28) and the results show no effect of Duox1 (**Figures 5G, H**). Additionally, tissue distribution of MPO inside the lung tissue was evaluated by immunohistochemistry (**Figure 6**). *Mtb*-infected lungs at 1 day post-infection exhibit undetectable MPO while high levels of MPO are evident inside inflammatory cell aggregates at 30 days post-infection in both WT and *Duox1* KO (**Figure 6A**) mice. Spatial NE expression inside the lung lesions of *Mtb*-infected mice revealed that NE also accumulates over time following MPO patterns independent of Duox1 (**Figure 6B**). These data demonstrate that release of the azurophilic granule content from neutrophils is not influenced by Duox1 during *Mtb* lung infection.

### Duox1-dependent B cells accumulate in the lung of *Mtb*-infected WT mice

**Figure 7:**
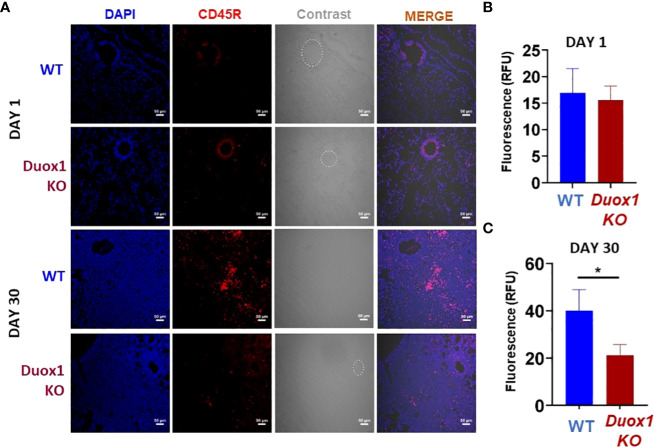
Duox1-depndent B cell accumulation in *Mtb*-infected lungs. *Duox1 KO* and WT mice were aerogenically infected with *Mtb* Erdman (200 CFU). Lung tissues were obtained at 1 and 30 day(s) post-infection, fixed and immunofluorescence staining was performed using anti-mouse CD45R specific for B cells (red). DAPI (blue) was used to depict cellular DNA. **(A)** Representative images of five similar results (n = 5 WT and n = 5 *Duox1* KO mice) are shown. Quantitation of CD45R fluorescence at **(B)** 1 day post-infection and **(C)** 30 days post-infection. Scale bars indicate 50 μm. Mean fluorescent intensity (MFI) ± SEM in arbitrary unit (AU) is shown (Mann–Whitney U test). Significance level is indicated as *, p < 0.05. *Duox1* KO, *Duox1*-deficient; WT, wild-type.

To further investigate a possible role for Duox1 in the immune responses against *Mtb*, we harvested the lungs of *Mtb*-infected WT and *Duox1* KO mice at 1 and 30 day(s) post-infection, and proceeded with immunofluorescence staining using a B cell-specific antibody (anti-mouse CD45R). Our results show that B cell numbers in the lung tissues (not BAL) at 1 day post-infection are the same between WT and *Duox1* KO mice (**Figures 7A, B**). However, at 30 days post-infection, the number of B cells is significantly higher in WT mice than in *Duox1* KO mice (p≤ 0.05) (**Figures 7A, C**). In contrast, B cell staining in the spleen at 30 days post-infection is comparable between *Mtb*-infected *Duox1* KO and WT animals (**Supplementary Figure 7**). When anti-*Mtb* IgG antibody titers were compared between *Mtb*-infected Duox1 KO and WT animals in different compartments (BAL, lung lysate, serum), no significant differences were found (**Supplementary Figure 8**). These results indicate that Duox1 may play a role in B cell trafficking but it is unlikely to affect overall B cell activation or function during *Mtb* infection in this mouse model.

### Duox1 suppresses caspase-3 activation in the lungs of *Mtb*-infected mice

**Figure 8:**
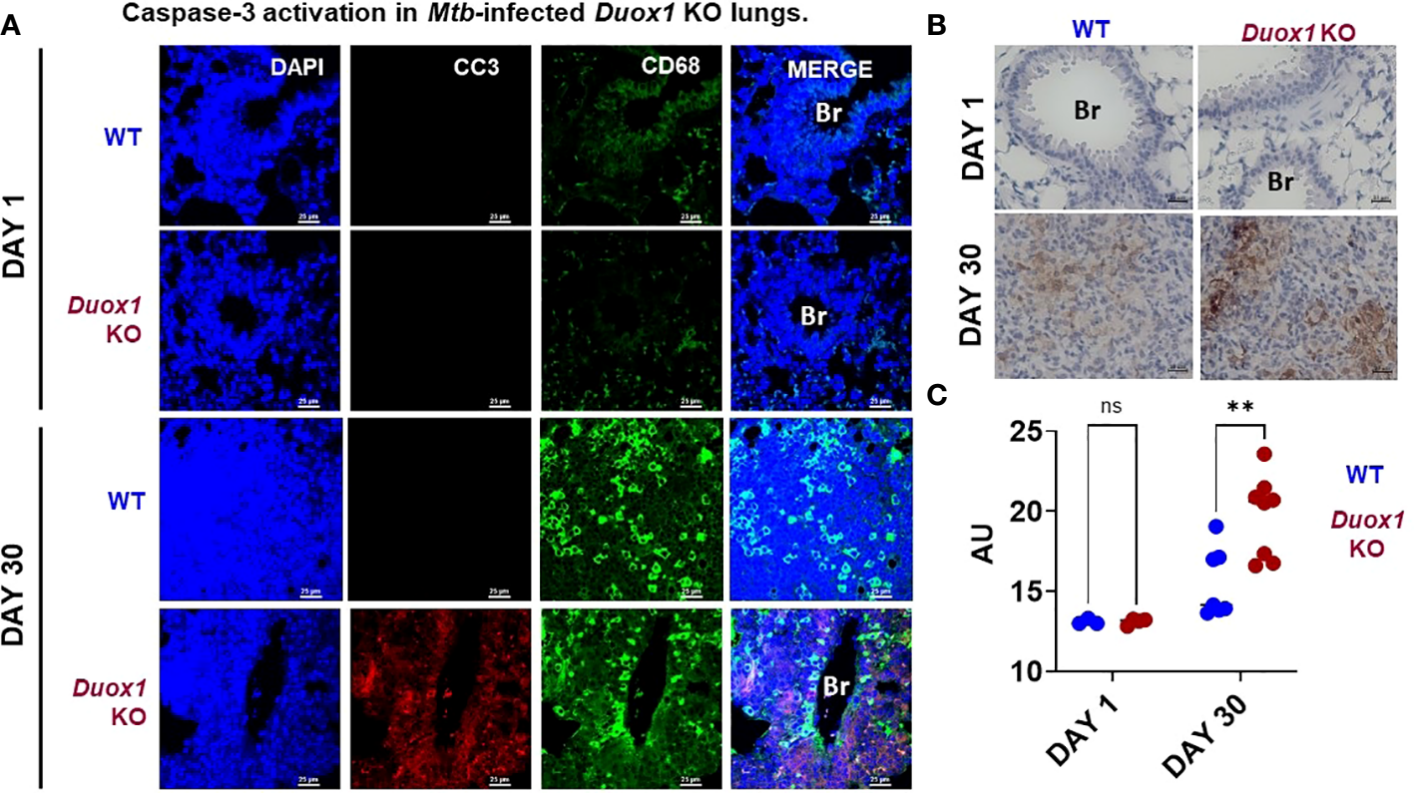
Caspase-3 activation in *Mtb*-infected *Duox1* KO lungs. *Mtb*-infected lungs of WT and *Duox1* KO mice were harvested and fixed at 1 or 30 day(s) post-infection. **(A)** Representative immunofluorescence staining of cleaved caspase-3 (CC3, red), CD68 (macrophages, green) and DAPI (DNA, blue). Scale bar: 25 µm). **(B)** Representative results of cleaved caspase-3 immunohistochemistry images at 1 (n=3 per genotype) and 30 days post-infection (n=8 per genotype). Scale bar indicates 25 µm. **(C)** Comparative non-stochiometric DAB signal quantification with ImageJ shows that the cleaved caspase-3 signal is significantly higher in the lung inflammatory aggregates of *Duox1 KO* mice compared to WT mice at 1 (n=3) and 30 (n=8) day(s) post-infection. A 2-way Anova with Sidak’s multiple comparison test was used for statistical data analysis. Significance levels are indicated as **P ≤ 0.01. AU, arbitrary unit; Br, bronchus; CC3, cleaved caspase-3; *Duox1* KO, *Duox1*-deficient; WT, wild-type.

Apoptosis plays a vital role in host defenses against pathogens. During *Mtb* infection, macrophage apoptosis can be beneficial to the host by activating innate and adaptive immune responses (37, 38). Cleaved caspase-3, the major executioner of apoptosis, degrades multiple proteins and is responsible for DNA fragmentation and morphological changes (39). Using immunofluorescence staining [as done previously (20)], we found significantly higher caspase-3 activation/cleavage in *Mtb*-infected *Duox1* KO mice compared to WT counterparts suggesting that *Duox1* KO mice are more susceptible to apoptosis while caspase 3 activation was undetectable at day 1 post-*Mtb* infection in both genotypes (**Figure 8A** and **Supplementary Figure 9**). Similar results were obtained using immunohistochemistry showing that cleaved caspase-3 expression is significantly higher in *Duox1* KO than in WT mice (*p* ≤ 0.05) (**Figures 8B, C**). No Duox1-dependent differences were observed in the tissue expression of the used macrophage marker, CD68 (**Figure 8A** and **Supplementary Figure 9**). Overall, these data indicate that Duox1 reduces apoptosis during *Mtb* lung infection.

## Discussion

DUOX1, an NADPH oxidase mainly expressed in bronchial epithelial cells, is the major source of airway epithelium-derived H_2_O_2_ (23). Because bronchial epithelial cells are one of the first lines of defense during respiratory infections, including TB, it was hypothesized that Duox1 will be protective against *Mtb* infection *in vivo*. Lactoperoxidase (LPO), an antimicrobial enzyme present in the airway, uses H_2_O_2_ to oxidize several substrates (15–18). LPO is abundant in the airway surface liquid of naïve mice (20). Its primary substrate, thiocyanate (SCN^-^) is also abundant in the airway secretions and can be converted by LPO into hypothiocyanite (OSCN^-^) that possesses antimicrobial properties against several pathogens *in vitro* (14, 21, 40). DUOX1 has been also shown to be expressed in human and murine macrophages which play a central role in *Mtb* infection in the lung in both species (30, 41, 42). Our primary goal was to assess whether Duox1 has a protective role against *Mtb* infection, given that DUOX1 is the main source of H_2_O_2_ in the airway. We have previously shown that DUOX1 has an important role in antiviral immunity *in vivo* by improving airway epithelial influenza viral clearance, shaping the early antiviral innate response, and reducing infection-related morbidity and mortality (20).

To extend these findings to other infectious diseases models, we assessed whether Duox1 prevents or limits *Mtb* infection, reduces or limits bacterial growth and dissemination, or eliminates or reduces lung pathology *in vivo*. Using a mouse model of aerosol infection with the strain *Mtb* Erdman, we addressed these primary objectives. The data show a dispensable role of Duox1 during *Mtb* infection *in vivo*. Neither bacterial growth in the lung, nor bacterial dissemination to different organs, such as the spleen and the liver, nor lung pathology were affected by Duox1 in this model. These data indicate that Duox1 does not have a protective role against *Mtb*, at least for the timeline (90 days post-infection) used as an endpoint in this study. Previous studies using a different strain of *Mtb* (H37Rv) have shown that mice start to show signs of illness and decreased survival beginning three months after infection (43), the time at which our mice were sacrificed. Additionally, previous studies have shown that *Mtb* has relatively high resistance to killing with H_2_O_2_.

Despite this, Duox1 had some significant effects in our *Mtb* model at three levels. First, *Duox1* KO mice showed significantly higher levels of certain cytokines/chemokines in the BAL fluid at 30 days post-infection as evidenced by higher concentrations of TNF-α, IL6, KC, CCL3, CCL2, CCL20, CXCL11, CCL11, CCL27, CXCL5, CXCL12, and CCL5. A similar, overall role of Duox1 in shaping the early cytokine environment of the airways was established in our prior study using an H1N1 influenza strain (20). While some of the observed differences in cytokine levels between *Mtb*-infected WT and *Duox1* KO mice are of interest, only four mice per group were used, therefore further experiments with more animals are required to confirm these data during *Mtb* infection. In contrast, our prior influenza study in *Duox1* KO mice used 19-20 mice per group for cytokine measurements (20). Duox1 deficiency did not affect the frequencies of lymphoid or myeloid cells at 30 and 90 days post-infection either. None of the neutrophil markers was different between WT and *Duox1* KO mice, suggesting that Duox1 is not required for neutrophil recruitment and activation in the lung environment during *Mtb* infection.

A previous study demonstrated that B cells infiltrate the granulomas of active *Mtb* patients (44) in accordance with earlier reports on the implication of B cells in TB pathogenesis (45, 46). Mechanistic studies assessing a possible role for B cells in *Mtb* infection used a mouse model with a moderately high dose of approximately 300 CFUs of the H37Rv strain (43). The study demonstrated that mice with altered B cell populations in the lung were more susceptible to aerosol infection than mice with normal B cells, suggesting that B cells can modulate immunity to *Mtb* infection (43). A prior study found that Duox1-derived H_2_O_2_ negatively regulates *in vitro* proliferative activity but not Ig isotype production in primary splenic CD19+ B cells in mice (47). Based on the results in our study, Duox1-dependent B cell infiltration in the lung is suggested during *Mtb* infection *in vivo.* However, this data was only derived from one method, immunofluorescence staining of a B cell-specific marker and secondary, more quantitative measurements would be required to strengthen the importance of this observation. Using B cell-deficient mice, the role of B cells in TB has been addressed in several studies (48–52). B cells are present in lymphoid clusters in mice (53), non-human primates (54) and human tuberculous granulomas (48, 55, 56). The function of B cells in mice lacking the ability to secrete immunoglobulin was uncovered by a study that supported the hypothesis that B cells can modulate immunity to *Mtb* in an organ-specific manner (43). Indeed, B cells participate in orchestrating cellular inflammatory aggregate formation during *Mtb* infection (50, 57). Our data are not sufficient to suggest a clinical or pathological role for B cells in TB in our model (58). Our results also show that the levels of *Mtb*-specific IgG levels are comparable between WT and *Duox1* KO mice. Previous studies have indicated that activation of B cells within the mucosa will generate mainly IgA-secreting plasma cells, while activation in lymph nodes will generate mainly IgG-secreting plasma cells that home preferentially to the bone marrow and provide systemic protection (58). However, recent evidence suggests that IL4-responsive B cells during chronic *Mtb* infection in mice have a perturbed antibody and inflammatory cytokines profile (59). Previous evidence also suggests that IgA+ B cells produce IL-10 in the gut (60) and during Mtb infection (44) but more detailed investigations are required to assess any relationship between B cells, anti-inflammatory responses, immunoglobulins, and cell death pathways such as apoptosis.

Finally, higher levels of cleaved caspase 3, a marker for apoptosis, in the lung of *Duox1* KO compared to WT mice may suggest more apoptotic cell death in *Mtb*-infected animals. Previous independent studies have shown the presence of apoptotic markers, including cleaved caspase-3, in murine and human lung following virulent *Mtb* infection (61–65). Apoptosis participates in host defense against *Mtb* facilitating cross-presentation of bacilli antigens contained in apoptotic bodies in the local lymph nodes (66), but its role in *Mtb*-infected *Duox1* KO mice is not addressed in this study. Previously, we showed a similar trend in an influenza infection model where the *Duox1* KO lung tissues (likely airway epithelium) showed higher levels of the same apoptotic marker (20). Unlike in the case of our data here on *Mtb*, Duox1 affected viral proliferation in the lungs of influenza-infected mice suggesting that Duox1 could play a pathogen-specific role in regulating apoptosis and general respiratory innate immunity.

## Study limitations

The study endpoint is short (90 days post-infection) for *Mtb* infection. Designing future experiments beyond this timeline and after 1 day post-infection may yield a more positive phenotype. Also, using GFP-labelled *Mtb* bacilli would have helped adding significant values to both the immunostaining on lung tissue sections and the multicolor flow cytometry. The sample size of the bioplex data generated in this study is small (n=4 per genotype per day post infection) and the cell origin of the cytokines/chemokines identified was not addressed. Another limitation is the lack of confirmation of the B cell immunostaining by flow cytometry that shows higher expression in *Mtb*-infected WT mice at 30 days post-infection compared to *Duox1*-deficient mice. B cells were originally omitted in the flow cytometry panel because investigating them in the BAL was not our initial goal. It was only later when we did not find significant differences for initial parameters that we assessed the levels of B cells in the lung. In this study, we have not addressed any possible interactions between memory B cells, immunoglobulins, anti-inflammatory cytokines, Duox1, and cell death.

Additionally, previous studies have demonstrated that *Mtb* bacteria resistant to isoniazid, a treatment for active TB, have deletions in the *katG* gene, which encodes for the enzyme catalase (67). Several pathogens promote the degradation of H_2_O_2_ through the presence of genes that encode for enzymes with catalase activity (68). Recent evidence suggests that *Mtb* catalase inhibits the formation of mast cell extracellular traps by degrading H_2_O_2_ (69). This could be studied further in the context of Duox1. There are differences in lung structure and TB pathogenesis between humans and mice that could not be accounted for in this work solely using a mouse model. Our study was performed in mice with global Duox1 deficiency. Hence, Duox1 deficiency in cells other than airway epithelial cells could have also played a role. While caspase-3 cleavage is widely used as an apoptosis marker, the use of an additional apoptosis assay, such as the TUNEL assay, would be required to confirm the Duox1-dependence of apoptosis during *Mtb* infection. The cell type with enhanced cleaved caspase-3 signal has not been identified. The cleaved caspase-3 signal largely does not overlap with the CD68 staining indicative of macrophages.

In conclusion, our work unravels that Duox1 does not play a major role in the immune response or pathogenesis of *Mtb* lung infection *in vivo*.

## Data availability statement

The original contributions presented in the study are included in the article/**Supplementary Material**, further inquiries can be directed to the corresponding author.

## Ethics statement

The animal study was reviewed and approved by Animal Care and Use Committee at the University of Georgia.

## Author contributions

BR, TG, and DS designed the experiments. TG, DS, KF, NA performed the experiments. DS, TG, KF, and BR analysed the data. KS performed the pathological evaluation. DS wrote the original draft. DS, TG, KF, NA, KS, FQ, and BR revised and edited the manuscript. FQ provided access to BSL3. BR and FQ received funding and oversaw the project. All authors contributed to the article and approved the submitted version.
