# Supplementary material for: Dual oxidase 1 is dispensable during Mycobacterium tuberculosis infection in mice
Source: Front Immunol. 2023 Mar 3;14:1044703. doi: 10.3389/fimmu.2023.1044703 (PMC10020924; doi:10.3389/fimmu.2023.1044703)

Supplemental Figure 1. The lung cytokine environment in Duox1-deficient and WT mice.

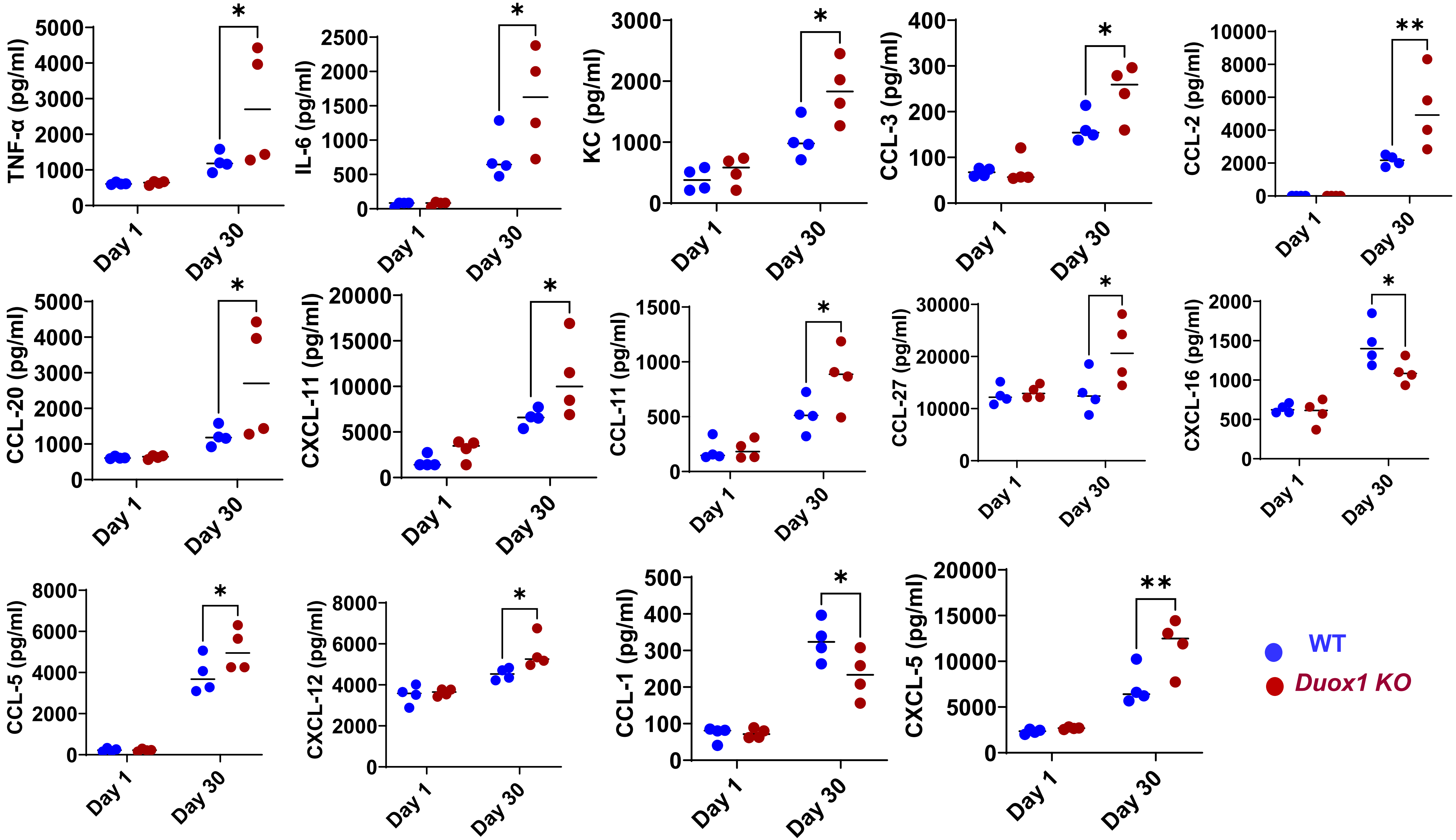

Supplementary figure 2. BAL levels of cytokines and chemokines unaffected by Duox1 in *Mtb* infection.

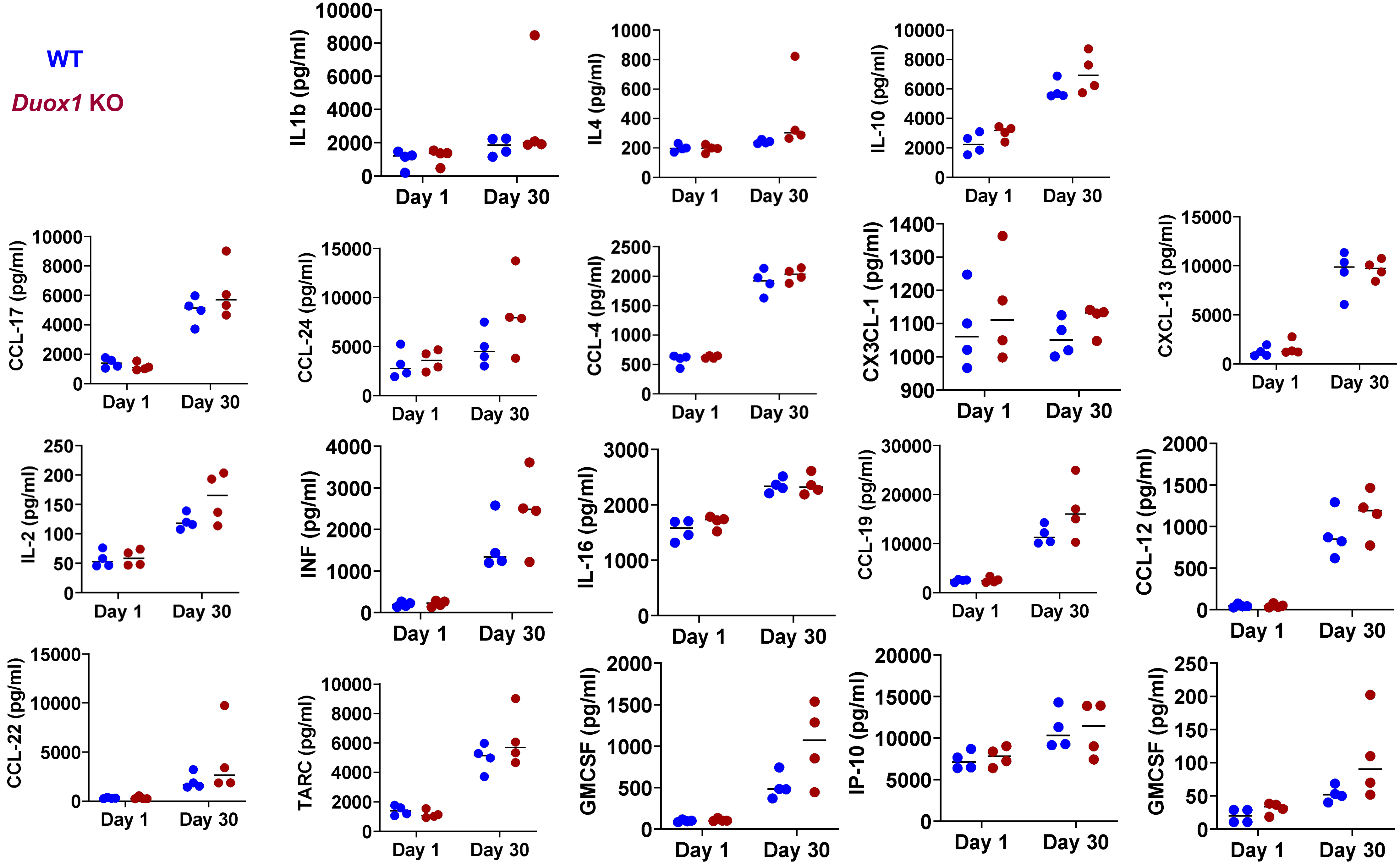

Supplementary figure 3. Myeloid cell clustering by uniform manifold approximation and projection (UMAP) in the lungs of *Mtb*-infected WT and *Duox1* KO mice.

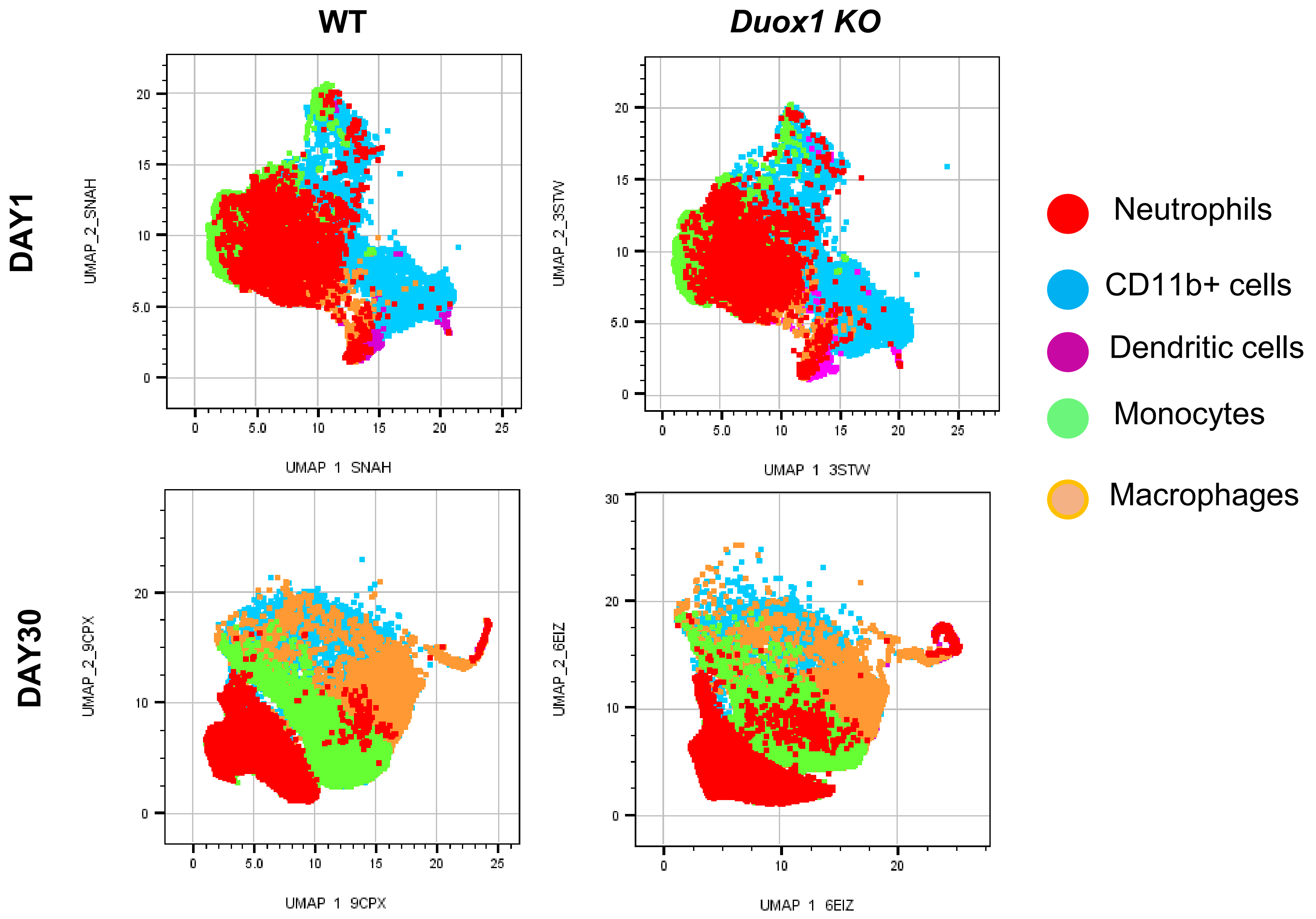

Supplementary figure 4. Similar lymphoid cell profiles in the spleens of *Mtb*-infected WT and *Duox1* KO mice.

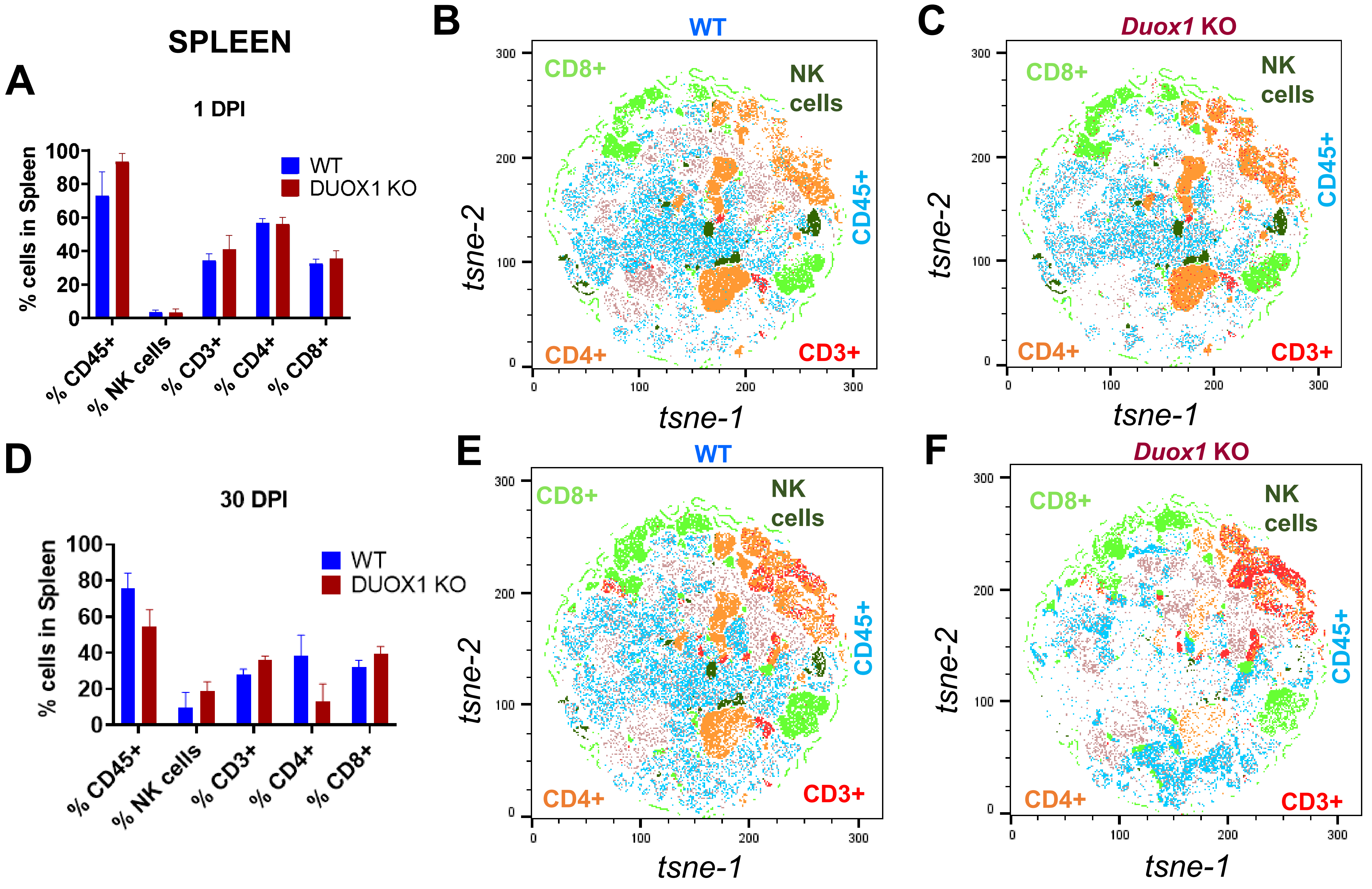

Supplementary figure 5. Distribution of myeloid cell profiles in the spleen of *Mtb*-infected WT and Duox1 KO mice.

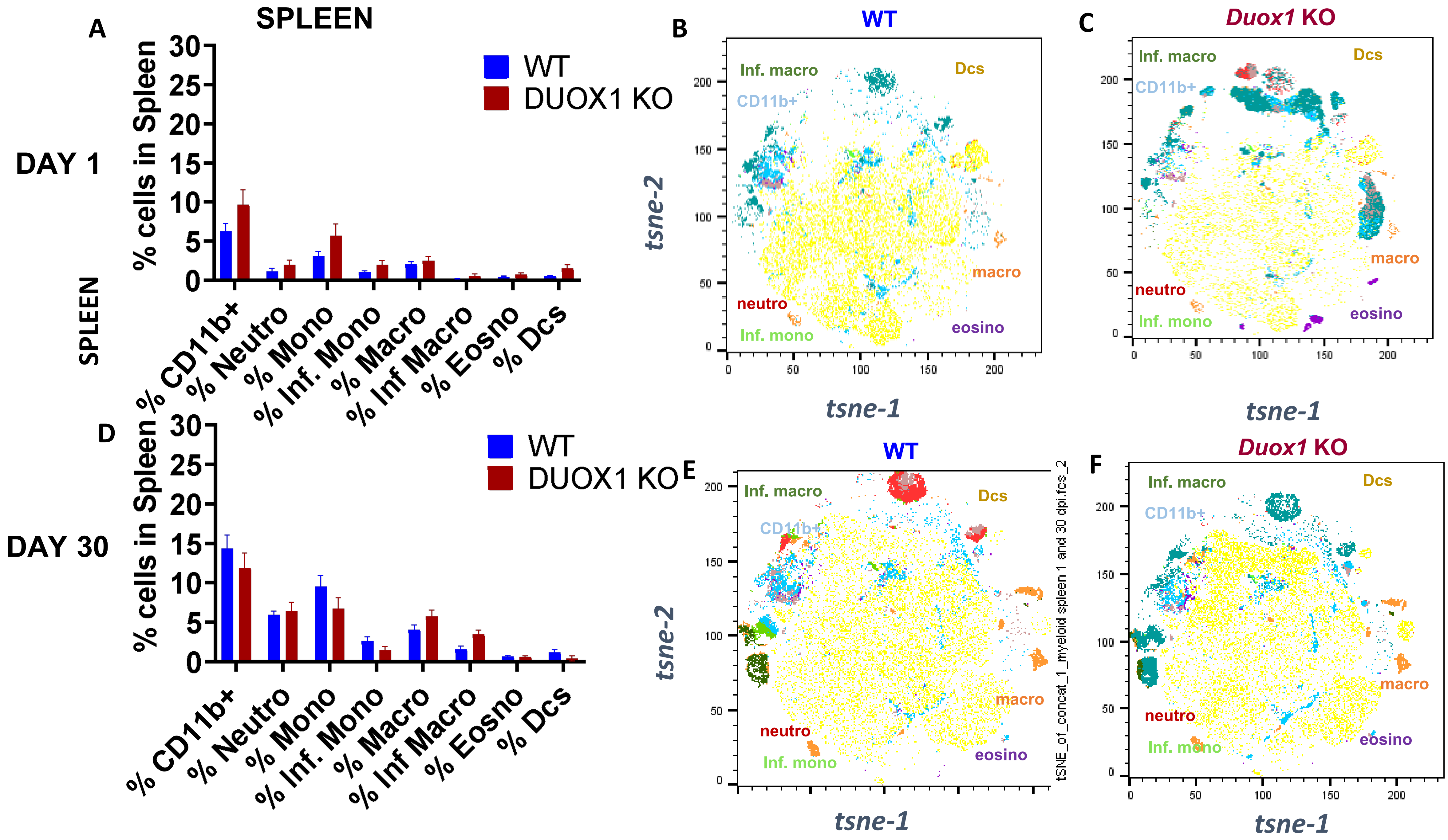

Supplementary figure 6. Myeloid cell populations in the BAL and MLNs of *Mtb*-infected WT and *Duox1* KO mice.

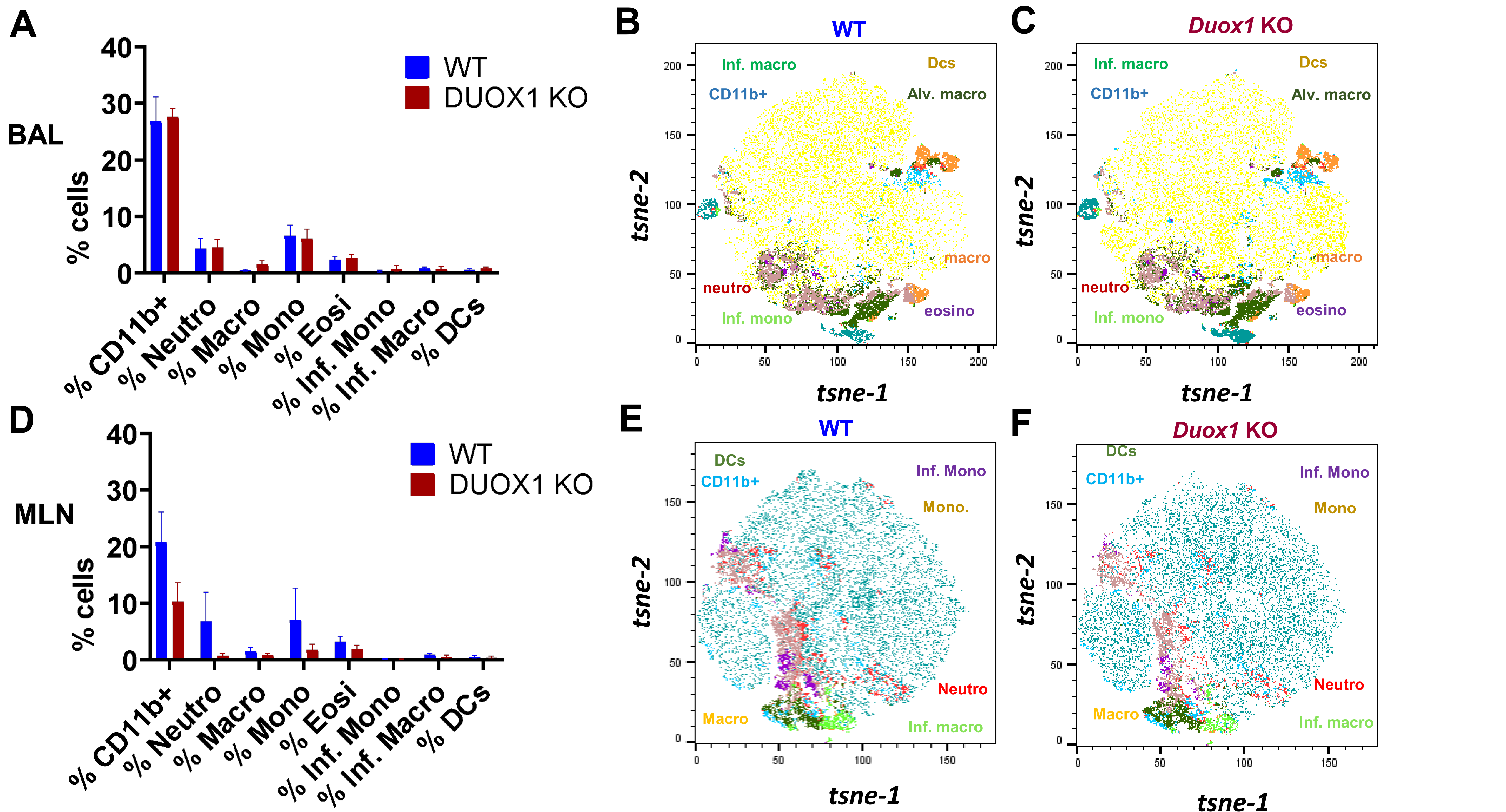

Supplementary Figure 7. B cells in the spleen of *Mtb*-infected *Duox1* KO and WT mice.

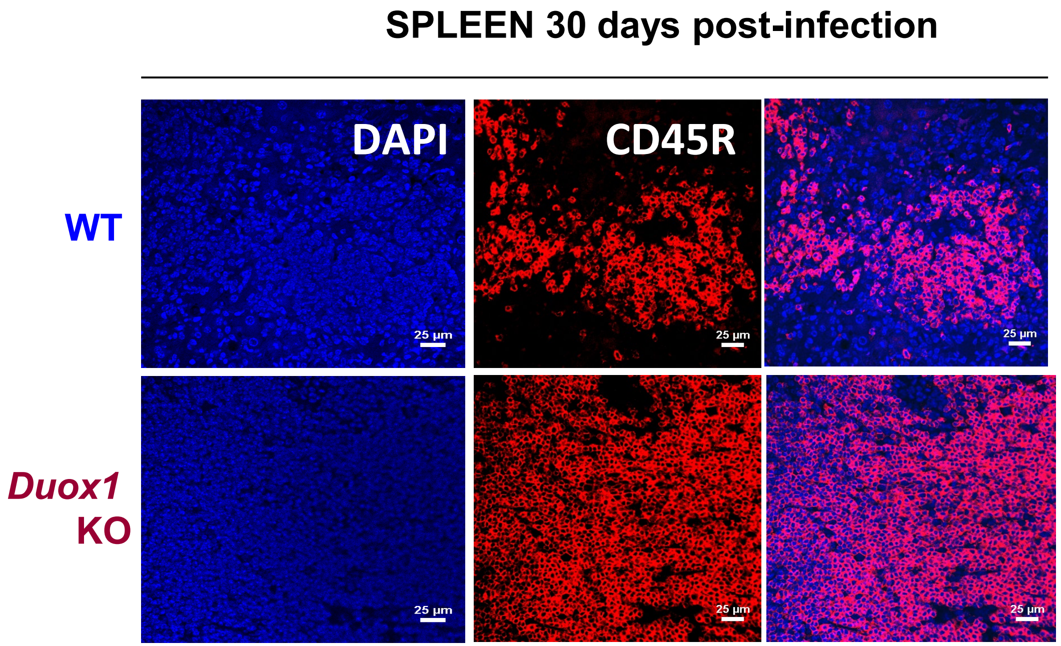

Supplemental Figure 8. ***Mtb*****-specific IgG titers are similar between *Duox1* KO and WT animals following *Mtb* infection.**

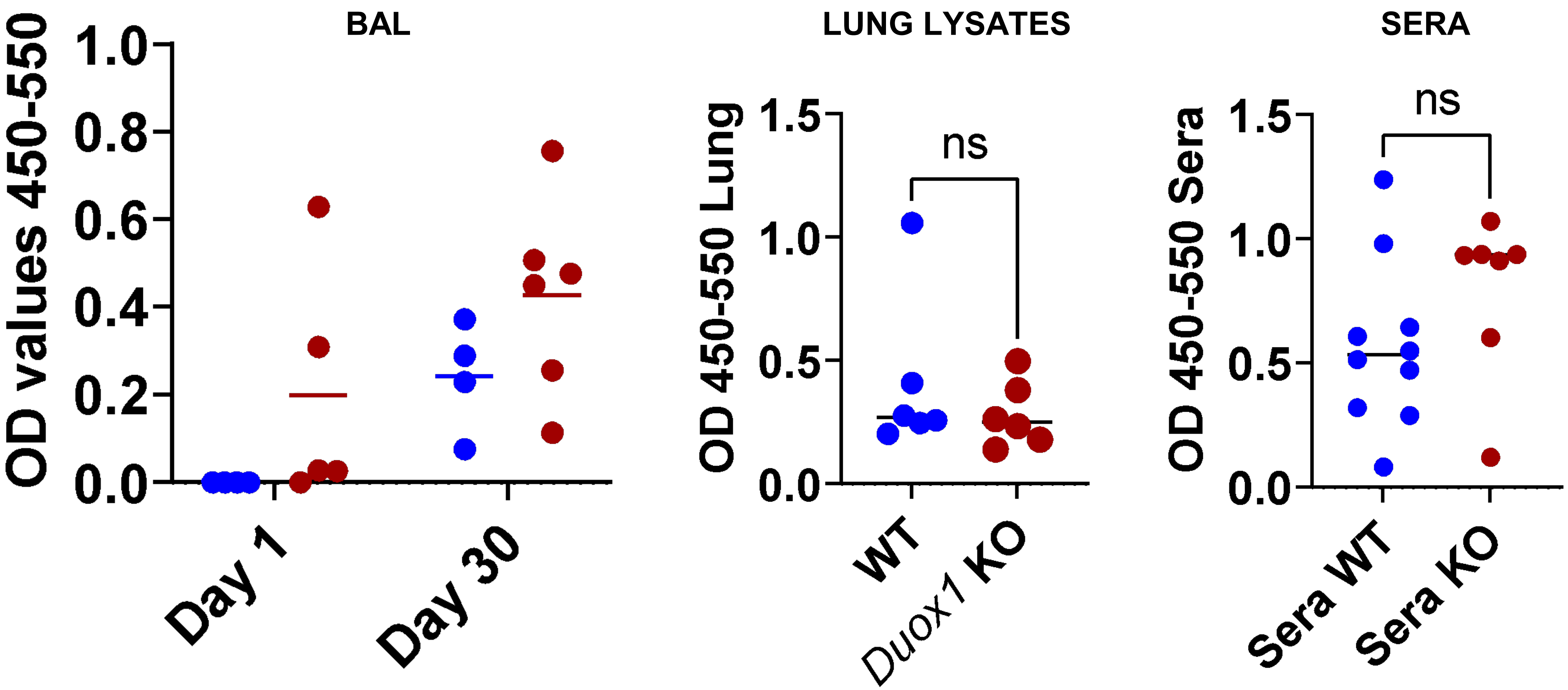

Supplementary Figure 9.
Quantification of CD68 and cleaved caspase-3 immunofluorescence values in *Mtb*-infected mice.

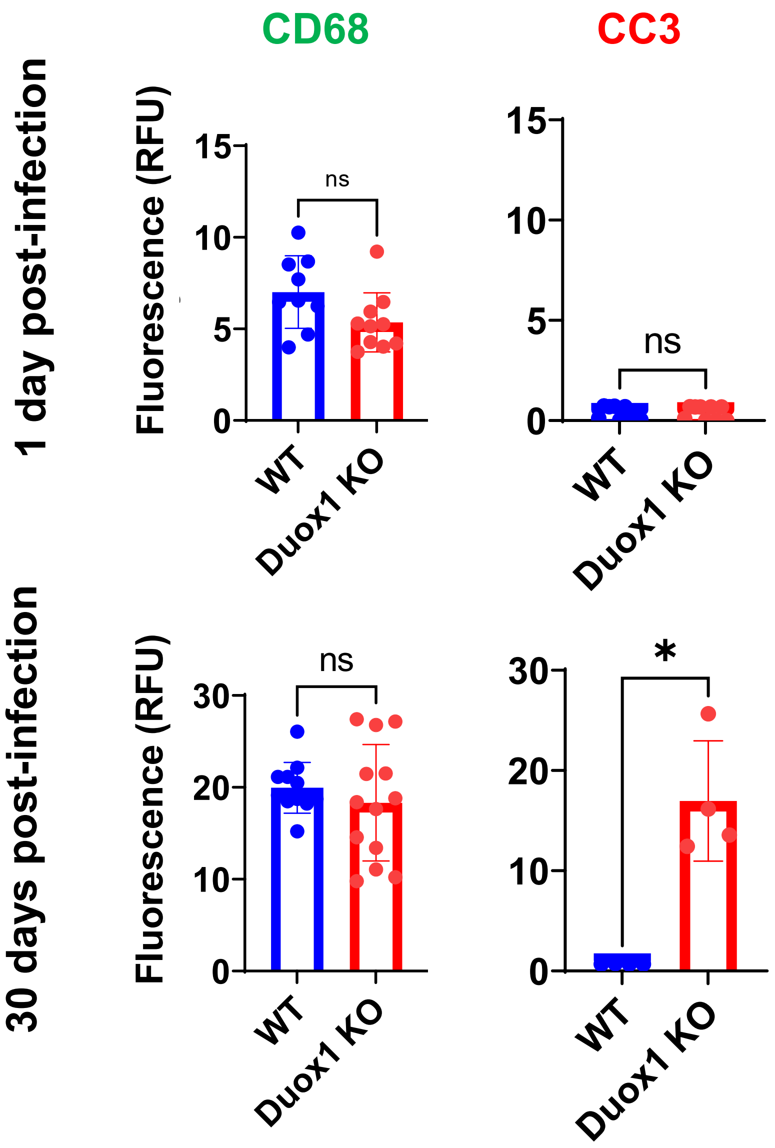

Supplement: Supplementary file 1 [file DataSheet_1.pdf]
